# N,N-Dimethylacetamide Exposure–Induced Acute Liver Failure

**DOI:** 10.1016/j.shaw.2026.03.010

**Published:** 2026-04-04

**Authors:** WonYang Kang, Eun Young Kim, Byung-Soon Choi

**Affiliations:** 1 Department of Research for Occupational Health, Institute of Occupation and Environment, Korea Workers' Compensation & Welfare Service, Incheon, Republic of Korea; 2 Department of Occupational and Environmental Medicine, Yeocheon Chonnam Hospital, Yeosu, Republic of Korea

**Keywords:** acute liver failure, chemically induced liver toxicity, fulminant hepatitis, toxic hepatitis

## Abstract

This study aimed to share the case caused by N,N-Dimethylacetamide (DMAC) exposure. We report a 30-year-old man who died from DMAC exposure–induced acute liver failure after working in the cutting and cleansing of hollow fiber membranes for 5 days. While he was exposed to low-level DMAC by inhalation as the measurement of periodic working environment monitoring, considering his working method, considerable DMAC exposure via dermal absorption was suspected. Therefore, short-term, short-latency DMAC exposure via dermal absorption can cause fatal acute liver failure owing to idiosyncratic reactions.

## 1 Introduction

N,N-Dimethylacetamide (DMAC), which is widely used as a solvent for the production of fibers, adhesives, and pharmaceuticals, has a toxic effect on human liver [1]. The exposure duration of DMAC-induced toxic hepatitis was reported in two cases from 2 weeks to 3 months in the production of acrylic fiber and seven cases from 6–15 weeks in the production of spandex fiber [2,3]. After following 440 new elastane fiber workers, there were 28 cases of DMAC-induced hepatic injury, and the median value of exposure duration was 1.5 months [4]. All cases of DMAC-induced toxic hepatitis mentioned recovered after stopping DMAC exposure and conservative treatment. Here, we present a case of DMAC-induced toxic hepatitis that worsened to acute liver failure and death by short duration of exposure and dermal exposure to DMAC.

## 2 Case report

### 2.1 Case presentation

On 22 June 2020, a 30-year-old man started to work in a company producing a hollow fiber membrane of water purifiers. The patient’s first department was the potting process. On 27 July 2020, his job was changed to cutting and cleansing a hollow fiber.

On 1 June 2020, the patient visited an outpatient clinic for tingling on the hand, and blood test showed a white blood cell count of 6,200/μL, aspartate aminotransferase (AST) level of 14 IU/L, alanine aminotransferase (ALT) level of 20 IU/L, gamma-glutamyl transpeptidase (γ-GTP) level of 20 IU/L, and total bilirubin level of 0.3 mg/dL. In a prejob medical examination on 24 June 2020, the patient had no symptoms, and physical examination revealed normal gastrointestinal tract. Blood test showed AST, ALT, and γ-GTP levels of 23, 26, and 13 IU/L, respectively.

On 3 August 2020, the patient visited an outpatient clinic for epigastric pain 7 days after being assigned the new job. Physical examination showed icteric sclera and dark urine. Blood test showed the following findings: white blood cell count: 9,880/μL; platelet count: 217,000/μL; hemoglobin: 16.3 mg/dL; blood urea nitrogen: 4.1 mg/dL; creatinine: 0.6 mg/dL; sodium: 139 mEq/L; chloride: 97 mEq/L; potassium: 4.7 mEq/L; AST: 2,953 IU/L; ALT: 4,626 IU/L; γ-GTP: 117 IU/L; alkaline phosphatase: 159 IU/L; lactate dehydrogenase: 2,160 IU/L; total bilirubin: 11.9 mg/dL; direct bilirubin: 2.6 mg/dL; amylase: 34 IU/L; lipase: 83 IU/L; and alpha-fetoprotein: 3.11 ng/mL. Urinalysis findings were normal. Abdominal ultrasonography showed a right hepatic lobe hypoplasia, collapsed gallbladder, and mild splenomegaly. Abdominal computed tomography was unremarkable, excluding fatty liver lesion. A doctor prescribed oral silymarin, ursodeoxycholic acid, and biphenyl dimethyl dicarboxylate for 7 days.

The patient was found dead in his home on 5 August 2020. Blood, suspected to be hematemesis, was observed around the patient’s mouth (Fig. 1).

**Fig. 1:**
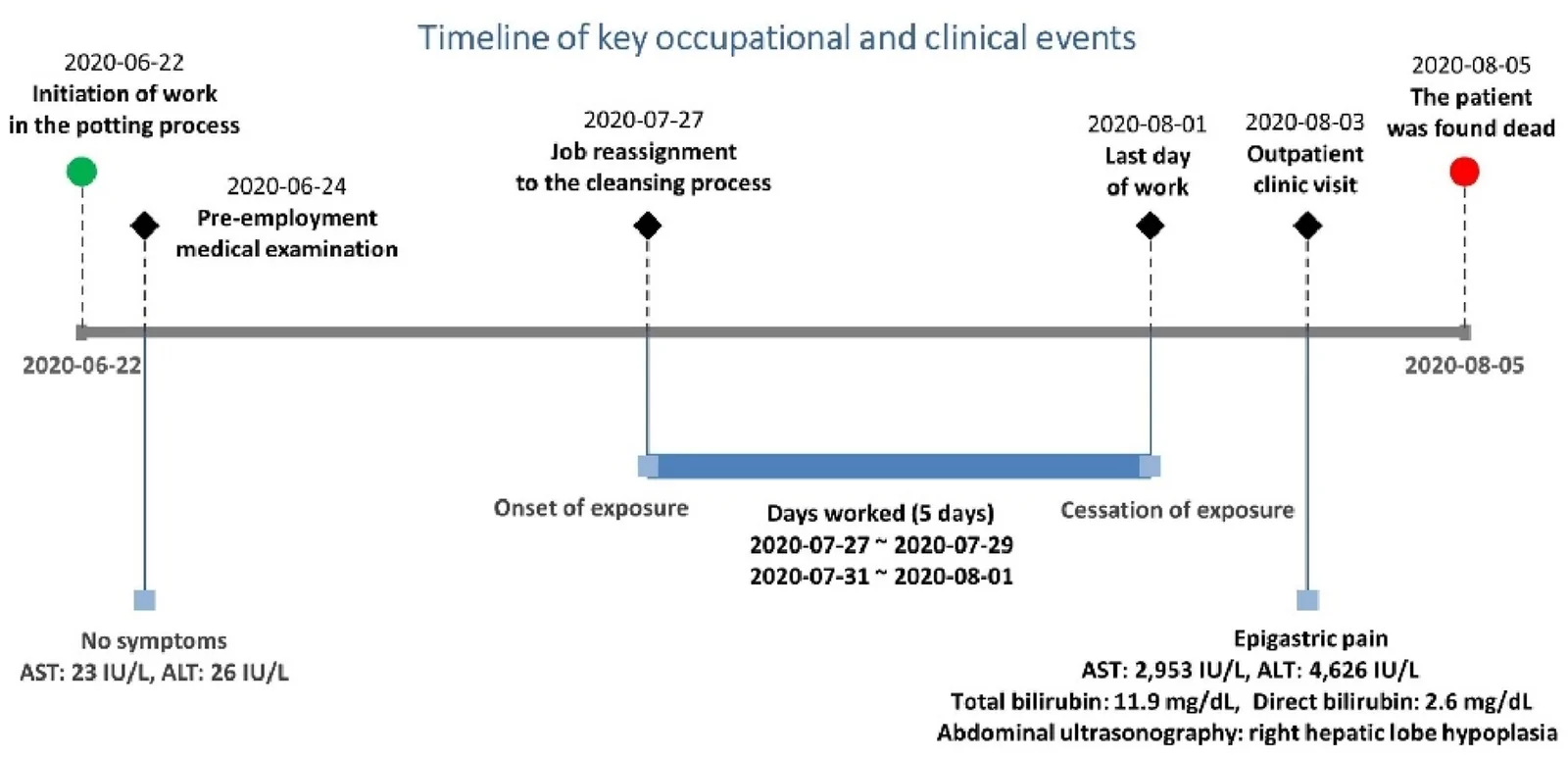
Timeline of key occupational and clinical events.

Autopsy was conducted and did not find evidences of injury, asphyxia, and poisoning leading to death. Autopsy reported extensive necrosis of hepatocyte, decreased volume and weight of the liver, icteric lesion in the eyes and brain, and petechial hemorrhage in the pericardium, mesentery, and subcutaneous soft tissue. The forensic pathologist could not find any cause of death in the whole body, excluding liver lesion; therefore, they concluded that liver failure may be the cause of death.

Patient consent form was provided by the bereaved family, and all procedures performed in studies were in accordance with the 1964 Helsinki Declaration and its later amendments or comparable ethical standards.

### 2.2 Occupational history

The process of producing a hollow fiber membrane is as follows: combination (solution make up) – spinning – cutting and cleansing – casing – potting (sealing) – exam – packaging. The patient had a full-time work of 10 hours/day and worked in the cutting and cleansing process for 5 days. The patient’s main job was cleansing, whereas cutting was an auxiliary job. The patient bundled hollow fiber up to the membrane after the spinning process and cut the membrane. The membrane was placed into the cleansing container manually. Cleansing for removing the residue on membrane used 50–60°C water. The patient removed the membrane from cleansed water and dewatered the membrane using a spin-dryer (Fig. 2).

**Fig. 2:**
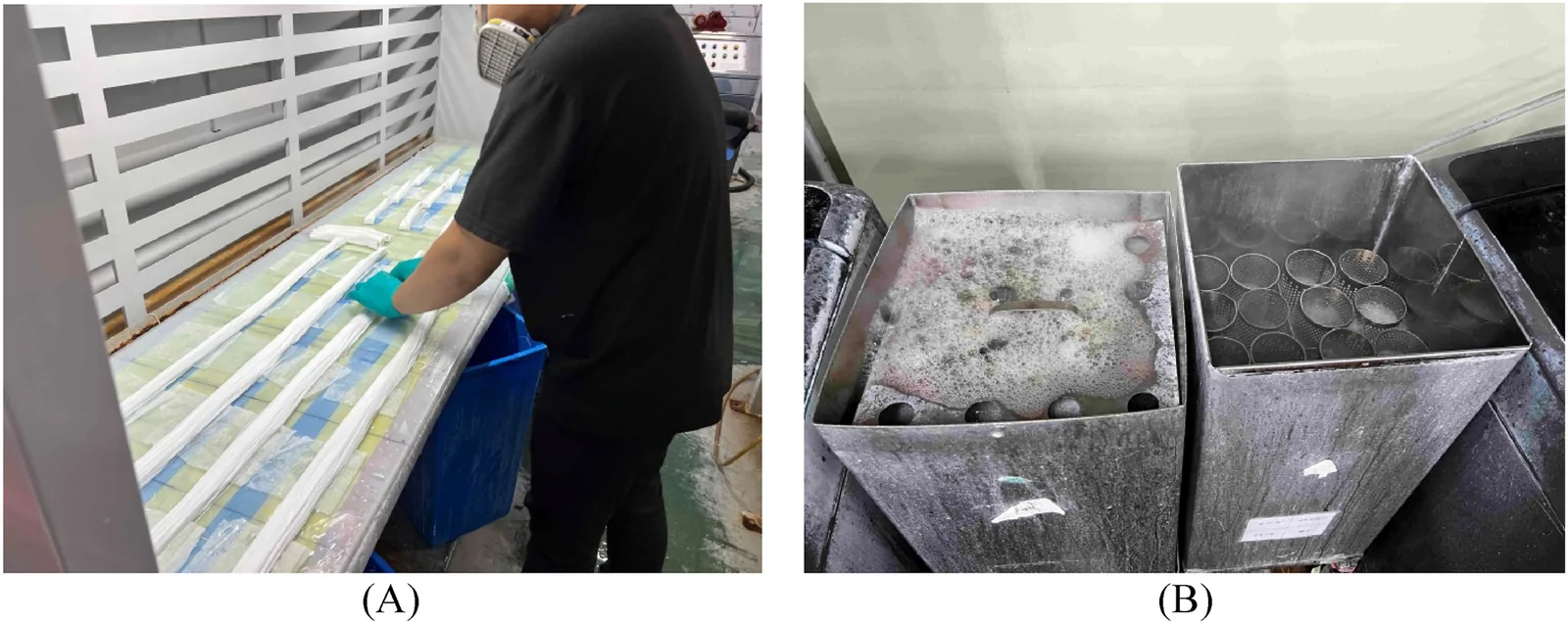
The patients work process. (A) The case bundled hollow fiber up to the membrane and cut the membrane. (B) The cleansing container, which the case put the membrane into and removed the membrane manually.

DMAC (Chemical Abstracts Service (CAS) Registry Number: 127-19-5) was used as a solvent combined with polysulfone, as a raw material, and other additive materials. As the measurement of periodic working environment monitoring from 2020 to 2023, the 16 samples were collected from workers equivalent to patient. The average mean of DMAC concentration was 2.36 ppm (range: nondetection to 6.00). The concentrations of DMAC in two samples were 5.37 ppm and 6.00 ppm, exceeding 50% of the Korean occupational exposure limit (10 ppm), and DMAC was not detected in two samples.

### 2.3 Differential diagnosis

The patient experienced epigastric pain and jaundice 2 days before death, and the levels of liver function test and bilirubin, which were normal 2 months before death, increased 10 times. Ultrasonography and autopsy revealed decreased liver volume, and autopsy confirmed the extensive necrosis of hepatocyte. Collectively, we concluded that the cause of death was acute liver failure induced by acute hepatitis.

We eliminated liver and bile duct diseases, such as liver cirrhosis, neoplasm, and cholangitis, through abdominal computed tomography and autopsy. We eliminated viral hepatitis by anti–hepatitis C virus antibody as negative and anti–hepatitis A virus immunoglobulin G antibody as positive tested approximately 2 years before death. Hepatitis B surface antigen and anti–hepatitis B surface antibodies were negative approximately 2 months before death, and the patient was vaccinated twice before death. Moreover, the patient had taken medicines, including itopride hydrochloride, omeprazole, and choline fenofibrate, approximately 2 months before death. However, liver function test was normal in the prejob medical examination, and drug concentration was detected within therapeutic dose range through autopsy. Thus, we excluded drug-induced hepatitis. The patient was a non-drinker, and ethyl alcohol and beta-hydroxybutyrate concentrations were normal through autopsy [5]. Therefore, we excluded alcoholic hepatitis. As the blood test for antinuclear antibody was negative tested approximately 2 years before death, autoimmune hepatitis was not the cause of acute liver failure (Table 1).

**Table 1:** Differential diagnosis toxic hepatitis from other liver diseases

Type of disease	Laboratory	Imaging	Autopsy	Remarks
Liver and bile duct diseases				
Liver cirrhosis, Neoplasm, Cholangitis		Abdominal CT(-): 2020-08-03	Unremarkable	
Acute viral hepatitis				
Hepatitis A	IgG anti-HAV(+): 2018-04-02			Protective antibody
Hepatitis B	HBs Ag(-), Anti-HBs(-): 2020-06-01			Hepatitis B vaccination: 2020-06-08, 2020-07-24
Hepatitis C	Anti-HCV(-): 2018-04-02			
Drug-induced hepatitis	AST/ALT/r-GTP, 23/26/13 (IU/L): 2020-06-24		Drug concentration: Therapeutic dose	Itopride hydrochloride, omeprazole and choline fenofibrate taken from 2020-06-01
Alcoholic hepatitis			Ethyl alcohol and β-hydroxy butyrate concentrations: Normal range	Non-drinker
Autoimmune hepatitis	ANA(-): 2018-04-02 RF(-): 2018-09-12		Incompatible with AIH histology	

AIH, autoimmune hepatitis; ALT, alanine aminotransferase; ANA, antinuclear antibody; AST, aspartate aminotransferase; CT, computed tomography; HAV, hepatitis A virus; HCV, hepatitis C virus; Ig, immunoglobulin.

## 3 Discussion

The patient could be much more exposed to DMAC via dermal absorption while cleaning a hollow fiber membrane by immersing hand in water for which the residual DMAC of fiber would be soluble.

In a study by Perbellini et al., although the measurement of environmental DMAC about synthetic acrylic fibers process was not >1.5 ppm, approximately 20% of the 223 workers’ urine concentration of N-methylacetamide (NMAC), a metabolite of DMAC, was >30 mg/g∙Cr. This suggests that DMAC can easily adhere to the skin, especially if wet [6].

In a study by Nomiyama et al., the mean urine concentration of NMAC after 4 hours at 6.1 ppm DMAC vapor exposure was 11.2 mg/g∙Cr [7]. The value of 6.1 ppm DMAC exposure in the experiment was similar to the maximum DMAC concentration which was 6.00 ppm in the patient’s workplace. According to the extrapolation to 10-hour exposure as the patient’s working hours, the estimated urine concentration of NMAC in the patient was assumed to be 20.4 mg/g∙Cr and 22.7 mg/g∙Cr.

However, this value of NMAC concentration reflects exposure to DMAC vapor via the lungs and skin. Direct skin exposure to residual DMAC dissolved in water during the cleansing process is not reflected in the aforementioned value. When the epidemiological survey was performed after the patient’s death, workers wore latex gloves covering their hands. Even if the patient wears gloves during cleansing, the cleansing container is deep and the gloves only protect the hands, leaving the forearm directly exposed to DMAC. In addition, wet working conditions during cleansing may enhance the dermal absorption rate of DMAC, as illustrated in Fig. 3. In conclusion, considering the exposure to DMAC vapor and direct skin exposure to DMAC, the estimated exposure level of DMAC may exceed 30 mg/g∙Cr in the NMAC urine concentration as the biological exposure index.

**Fig. 3:**
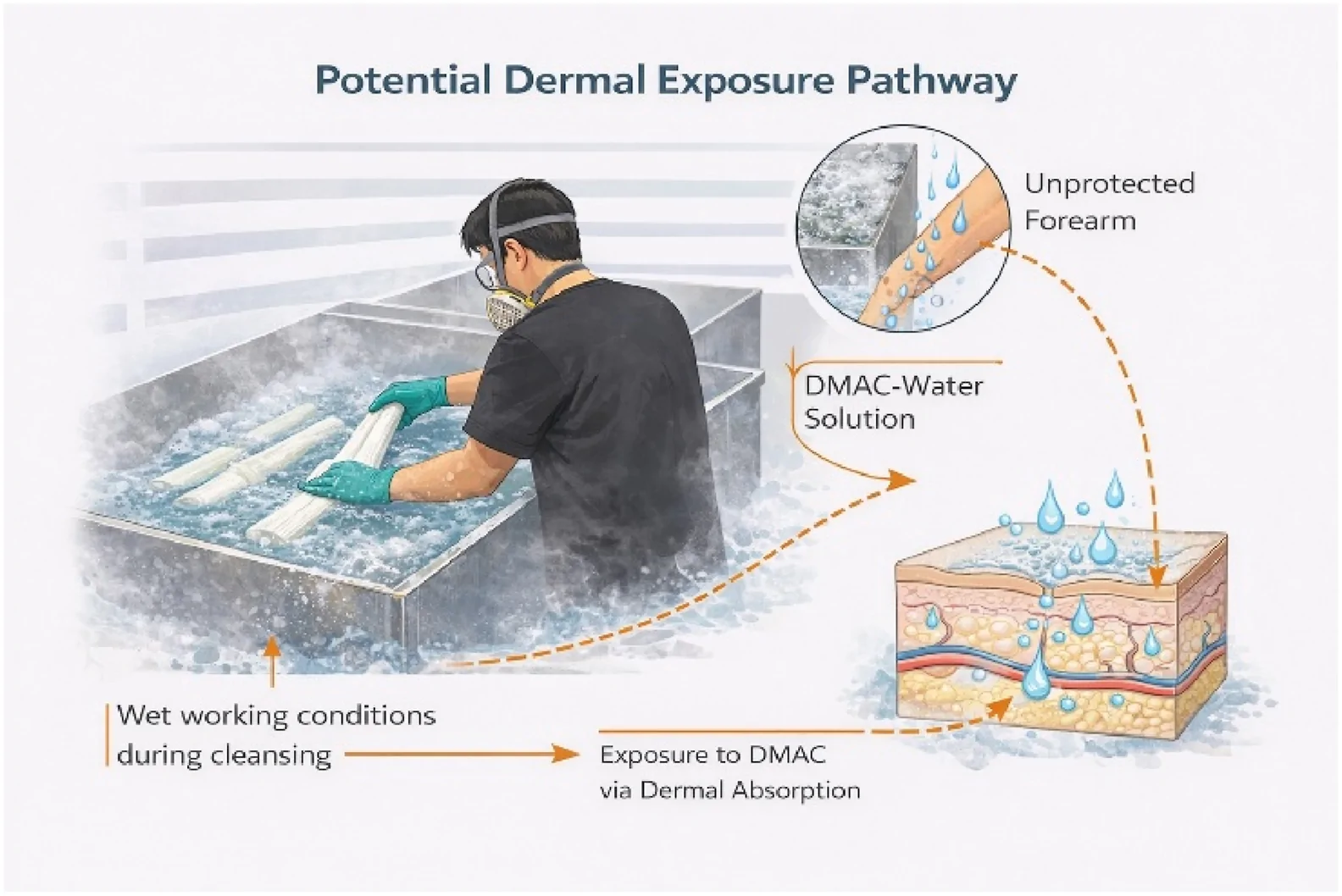
Schematic illustration of the hypothesized dermal exposure pathway to N,N-dimethylacetamide (DMAC).

Collectively, the other cause of acute hepatitis can be excluded. The case was exposed to DMAC 7 days before the onset of symptoms, and the patient might be exposed to considerable DMAC mainly by dermal absorption for 5 working days. Therefore, it was reasonable to diagnose this case as DMAC-induced toxic hepatitis worsening to acute liver failure leading to death.

This case has two unusual points, which are short latency and fast progression to acute liver failure attributable to the unpredictable trait of idiosyncratic reaction. Idiosyncratic reactions, which are thought to involve immunologic injury and direct hepatotoxicity related to individual variability in the generation of toxic metabolites, are generally recognized as unpredictable and characterized by a variable clinical course; unlike direct toxic reactions, they are not clearly dose dependent [8]. The shortest latency of DMAC-induced toxic hepatitis reported was 9 days [9]. Idiosyncratic drug-induced liver injury generally evolves 7–14 days after the first exposure, and the shortest latency reported was approximately 2–4 days [10]. This case suggests that factors such as a short duration of exposure (5 days) to DMAC and short latency (7 days) can cause acute liver failure, leading to death.

Regarding risk management, the job, such as cleansing, should be changed to an automatic process, minimizing dermal exposure to DMAC. In the Republic of Korea, health examinations for DMAC exposure, including liver function tests, are required within 1 month after the first exposure to DMAC. However, the current system is insufficient for preventing toxic hepatitis. Therefore, in addition to routine health examinations, maintaining clinical vigilance for occupational exposure–related hepatotoxicity, promptly transferring patients with markedly elevated liver enzyme levels (e.g., >4,000 IU/L) to facilities capable of acute management, and ensuring that occupational exposure history does not delay urgent medical decision-making are essential strategies to prevent the progression of toxic hepatitis.

This study has a limitation. Exposure assessment was estimated based on airborne measurements, relevant literature, and working conditions, rather than on biological monitoring data from the patient or coworkers. Therefore, this study cannot explicitly determine the fatal hepatotoxic level of DMAC exposure, which likely occurred primarily via dermal absorption.

## CRediT authorship contribution statement

**WonYang Kang:** Writing – original draft, Investigation, Data curation, Conceptualization. **Eun Young Kim:** Investigation, Formal analysis. **Byung-Soon Choi:** Writing – review & editing, Investigation.

## Statement on the use of AI

During the preparation of this manuscript, the authors used ChatGPT to assist in creating Figure 3. All outputs were reviewed and edited by the authors, who take full responsibility for the final manuscript.

## Conflicts of interest

The authors have no competing interests to declare.
